# Distribution of neurons in functional areas of the mouse cerebral cortex reveals quantitatively different cortical zones

**DOI:** 10.3389/fnana.2013.00035

**Published:** 2013-10-21

**Authors:** Suzana Herculano-Houzel, Charles Watson, George Paxinos

**Affiliations:** ^1^Instituto de Ciências Biomédicas, Universidade Federal do Rio de Janeiro, Cidade UniversitáriaRio de Janeiro, Brazil; ^2^Instituto Nacional de Neurociência Translacional, MCT/CNPqSão Paulo, Brazil; ^3^Faculty of Health Sciences, Curtin UniversityPerth, WA, Australia; ^4^Neuroscience Research AustraliaSydney, NSW, Australia; ^5^The University of South WalesSydney, NSW, Australia

**Keywords:** mouse, visual cortex, occipital cortex, cortical development, neuronal density, numbers of neurons

## Abstract

How are neurons distributed along the cortical surface and across functional areas? Here we use the isotropic fractionator (Herculano-Houzel and Lent, 2005) to analyze the distribution of neurons across the entire isocortex of the mouse, divided into 18 functional areas defined anatomically. We find that the number of neurons underneath a surface area (the N/A ratio) varies 4.5-fold across functional areas and neuronal density varies 3.2-fold. The face area of S1 contains the most neurons, followed by motor cortex and the primary visual cortex. Remarkably, while the distribution of neurons across functional areas does not accompany the distribution of surface area, it mirrors closely the distribution of cortical volumes—with the exception of the visual areas, which hold more neurons than expected for their volume. Across the non-visual cortex, the volume of individual functional areas is a shared linear function of their number of neurons, while in the visual areas, neuronal densities are much higher than in all other areas. In contrast, the 18 functional areas cluster into three different zones according to the relationship between the N/A ratio and cortical thickness and neuronal density: these three clusters can be called visual, sensory, and, possibly, associative. These findings are remarkably similar to those in the human cerebral cortex (Ribeiro et al., 2013) and suggest that, like the human cerebral cortex, the mouse cerebral cortex comprises two zones that differ in how neurons form the cortical volume, and three zones that differ in how neurons are distributed underneath the cortical surface, possibly in relation to local differences in connectivity through the white matter. Our results suggest that beyond the developmental divide into visual and non-visual cortex, functional areas initially share a common distribution of neurons along the parenchyma that become delimited into functional areas according to the pattern of connectivity established later.

## Introduction

How are neurons distributed across the surface of the cerebral cortex, and how does this distribution compare across functional areas as diverse as those that process specific sensory signals or associate information from various modalities? The mammalian cerebral cortex has traditionally been considered a homogeneous structure, with a constant number of neurons per surface area (N/A) across both cortical areas and species (with the exception of primary visual cortex; Rockel et al., 1980). Accordingly, such homogeneity has been assumed in most models of cortical development and evolution (Rakic, 1988; Prothero, 1997; Zhang and Sejnowski, 2000; Karbowski, 2003). However, recent quantitative studies by our group and others have shown that the N/A ratio is neither homogeneous across primate species (Herculano-Houzel et al., 2008), nor homogeneous across the cortical surface of primates (Collins et al., 2010; Cahalane et al., 2012). The latter studies found the largest neuronal densities, which indicate the smallest average neuronal sizes, in the posterior, visual areas of the cerebral cortex, and the smallest neuronal densities, which indicate the largest average neuronal sizes (including all dendritic branches), in the frontal-most areas of primate cortex. These differences in neuronal density are compatible with regional differences in dendritic structure found in the mouse cerebral cortex (Benavides-Piccione et al., 2006) and in the primate cortex (Bianchi et al., 2012). Such differences are also compatible with the sensory function of the visual areas, which relies heavily on local computation, and the associative function of the frontal-most areas, which requires massive integration of information from across the cerebral cortex.

Those studies on the heterogeneity of neuronal densities in the cortex, however, did not examine how neurons are distributed across the different functional areas that compose the cerebral cortex, nor did they determine whether the difference in neuronal densities across areas reflect area-specific rules or simply local variations of an underlying common rule that determines how neurons are added to the cerebral cortex.

Examining the distribution of neurons across functional areas requires well-established criteria for identifying and isolating these areas. Such criteria have been established in the most widely used mouse brain atlas, in which the cerebral cortex has been segmented by careful comparison of cytoarchitectonic, connectivity, and functional data (Franklin and Paxinos, 2007). The availability of these cortical subdivision maps, together with the small brain size, makes the mouse cerebral cortex an excellent structure for a first investigation of how neurons are distributed across functional areas. Recently, we estimated the total number of neuronal and non-neuronal cells that compose the mouse cerebral cortex at an average of 13.7 million neurons and 12.1 million other cells in the Swiss mouse (Herculano-Houzel et al., 2006). That study, however, did not address how neurons were distributed across functional areas.

The distribution of neurons across the surface of cerebral cortex is of functional interest, given that the number of neurons is likely to contribute to the capabilities of each functional area (Leingärtner et al., 2007), but it is also of considerable evolutionary and developmental interest. While the expansion of the cerebral cortex in evolution was for a long time considered to result mostly from the lateral addition of cortical columns resulting from increases in the number of precursors in the proliferating ventricular zone (Rakic, 1988), several recent studies have shown that lateral migration of interneurons from the ganglionic eminences, as well as expansion of the progenitor populations within the subventricular zone, are also likely to play a role in building cortices of different sizes (Kriegstein et al., 2006). All these are mechanisms whose regulation is likely to contribute to the number of neurons ending up composing individual cortical areas of different connectivity patterns and, therefore, function.

Within the cerebral cortex, different zones are specified very early in development through the action of morphogens and transcription factors (Bishop et al., 2000; Fukuchi-Shimogori and Grove, 2001; O'Leary et al., 2007), and these could in principle combine to specify particular functional areas and/or interact with other organizing factors such as thalamocortical axons (Dehay and Kennedy, 2007). It is possible that these factors influence neuronal proliferation and differentiation locally leading to combinations of numbers of neurons and average neuronal cell size that are particular to each functional area. An alternative possibility is that, in the other extreme, there may be a single, common mechanism that governs the distribution of neurons across the cortical surface, such that a single relationship between the volume of functional areas and their numbers of neurons are found (Cahalane et al., 2012). Indeed, local differences in numbers of neurons and neuronal density found along the non-human primate cerebral cortex (Collins et al., 2010) have been proposed to result from a single mechanism of formation of the cortical sheet, according to the timing of the balance between neuronal proliferation and differentiation (Finlay et al., 1998; Cahalane et al., 2012). Such gradients of neurogenesis have been found across the cerebral cortex of the ferret (McSherry and Smart, 1986; Noctor et al., 1997). In this scenario, all functional areas of the cerebral cortex should share a common relationship between their volume and the number of neurons they comprise.

While the rules governing the relationship between the size of functional areas and their respective numbers of neurons may or may not be particular to each individual area, or at least to larger cortical zones, we suspect that non-neuronal cells are added to the cerebral parenchyma in a universal manner, with comparatively invariant neuronal cell sizes. As a result, the numeric ratio between number of other cells and neurons (the O/N ratio) varies uniformly with neuronal density across structures (Herculano-Houzel et al., 2006; Herculano-Houzel, 2011).

Here we use the isotropic fractionator (Herculano-Houzel and Lent, 2005) to analyze the distribution of neurons across 18 areas of the isocortex of C57Bl/6J mice in order to compare the relationship between gray matter volume and number of neurons across areas, and to determine whether the O/N ratio indeed varies uniformly with neuronal density across structures.

## Materials and methods

We analyzed the cortex of four male C57Bl/6J mice, aged 6 weeks and with similar body masses of ~20 g. Animals were perfused transcardially with cold saline (0.9%) followed by 4% paraformaldehyde in phosphate buffer (PB). The fixed brain was immediately removed from the skull, and post-fixed by immersion in 4% paraformaldehyde for 3 days. For each animal, one hemisphere had the cerebral cortex (defined as all cortical areas lateral to the olfactory tract, minus the hippocampus) dissected whole from the rest of the brain, and total numbers of cells counted; while the other hemisphere was cryoprotected for 3 days in a 30% sucrose solution in PB, embedded in 6% gelatine, and cut into 200 μm coronal sections.

Thick coronal sections of cortex were then lightly stained to improve contrast between the gray and white matter by immersion in cresyl violet solution for a few seconds. The gray matter of each 200-μm section was then dissected into functional areas according to anatomical criteria, under a dissecting scope, by matching each section to the corresponding plane in the mouse brain atlas of Franklin and Paxinos (2007). For each mouse, the dissected pieces of tissue parts from each of the 18 cortical areas listed in Table 1 were pooled across the sections and processed together.

**Table 1:** **Distribution of neurons across cortical areas in the mouse brain**.

**Area**	**Areas in atlas**	**% cortical area**	**% cortical volume**	**Neurons**	**% cortical neurons**	**N/mm^2^**	**N/mm^3^**	**Other cells**	**T (mm)**
Infralimbic	PrL, IL, DP	3.05	2.40	114,397 ± 13,883	2.27	44,851	85,755	178,738 ± 7,179	0.523
Cingulate	Cg1, Cg2	4.69	3.64	155,489 ± 3,907	3.08	39,622	76,747	257,370 ± 4,170	0.516
Retrosplenial	RSD, RSGa-c	9.35	5.77	314,761 ± 24,802	6.23	40,210	98,148	613,762 ± 25,772	0.410
Parietal	LptA, MptA, PTPR	1.01	1.18	50,395 ± 7,452	1.00	59,653	76,588	75,880 ± 8,371	0.779
Motor	M1, M2	9.12	12.23	508,471 ± 37,133	10.07	66,598	74,775	613,176 ± 74,417	0.891
Frontal	FrA, Fr3	3.69	3.49	129,669 ± 32,437	2.57	42,013	66,771	154,181 ± 27,236	0.629
V2M	V2ML, V2MM	3.44	2.71	204,924 ± 31,466	4.06	71,218	135,801	258,318 ± 8,706	0.525
V1	V1, V1M, V1B	6.27	5.51	475,913 ± 68,484	9.43	90,650	155,426	519,939 ± 46,768	0.583
V2L	V2L	2.49	2.51	199,886 ± 41,511	3.96	96,081	143,185	203,510 ± 23,843	0.671
S1-limb	S1FL, S1HL, S1Sh, S1Tr	3.76	5.74	350,642 ± 66,822	6.95	111,319	109,919	403,580 ± 72,205	1.013
S1-face	S1J, S1Ulp, S1DZ, S1BF	9.62	13.92	673,919 ± 75,756	13.35	83,689	87,115	780,051 ± 79,283	0.961
S2	S2	3.68	8.57	227,763 ± 64,321	4.51	73,949	82,433	286,834 ± 32,157	0.897
Auditory	AuD, AuI, TeA, AuV	5.70	4.97	377,362 ± 19,018	7.47	79,178	109,730	398,397 ± 27,006	0.722
Insula	AI, GI, DI, AIP, AID, AIV	5.30	6.19	256,495 ± 64,959	5.08	57,800	75,506	354,728 ± 34,421	0.765
Orbital	LO, MO, VO	5.18	6.11	107,179 ± 17,289	2.14	24,891	48,109	178,333 ± 28,740	0.517
Ectorhinal	EcT, PRh	3.94	4.04	147,179 ± 20,248	2.92	44,632	69,066	234,016 ± 21,098	0.646
Entorhinal	DLEnt, DIEnt, MEnt, VIEnt	8.20	3.83	400,019 ± 36,262	7.92	58,303	100,130	574,736 ± 33,256	0.582
Piriform	Pir, CxA, Apis, BLP	11.50	7.19	353,592 ± 49,458	7.00	36,735	74,253	554,696 ± 54,606	0.495
Total		83.864 mm^2^	55,591 mm^3^	5,048,837 ± 412,123 neurons				6,640,234 ± 244,643 other cells	
Motor		9.12	12.23	508,471	10.07				
Sensory		51.76	57.31	3,120,496	61.81				
Visual		12.20	10.73	880,723	17.45				
Somatosensory		17.06	28.23	1,252,324	24.81				
Auditory		5.70	4.97	377,362	7.47				
Insular		5.30	6.19	256,495	5.08				
Piriform		11.50	7.19	353,592	7.00				

Numbers are corrected to compensate for losses due to sectioning and thus reflect total numbers per functional area in one cortical hemisphere ± standard error of the mean.

To determine total numbers of cells and of neurons across functional areas we used a modification for small volumes of the isotropic fractionator (Herculano-Houzel and Lent, 2005). The isotropic fractionator involves dissociating the fixed tissue of interest into a suspension of free, intact cell nuclei that can be stained with the DNA marker DAPI (4′, 6-diamidino-2-phenylindole, Invitrogen, USA) and counted at the fluorescent microscope with a coefficient of variation across aliquots of the same sample of typically 0.10 or less (Herculano-Houzel and Lent, 2005). Subsamples are then stained with anti-NeuN antibody (Millipore ab377, 1:200) and a suitable fluorescent secondary antibody, and scored for the determination of the percentage of DAPI-stained nuclei that are also positive for NeuN immunoreactivity (that is, nuclei that are neuronal). Numbers of non-neuronal nuclei, heretofore referred to as other cells (O), are determined by subtraction. Details of the method have been described elsewhere (Herculano-Houzel, 2012a,b). The modification of the method for processing small volumes consists of homogenizing the tissue (in this case, a collection of pieces of coronal sections of cerebral cortex) in a 1 ml glass homogenizer (Wheaton, USA) in a total volume of 500 μl of dissociation solution containing DAPI, and collecting aliquots for counting directly from this volume in the homogenizer, without any washes or other changes in volume.

The sectioning of the cerebral cortex into 200 μm sections and the dissection into different areas leads to loss of tissue that is only noticeable once total numbers of cells are tallied and compared across the sectioned and unsectioned hemispheres of each mouse. To report numbers of cells that correspond to the total for an unsectioned hemisphere, we corrected all numbers pertaining to individual functional areas by multiplying them by the ratio between the total number of cells in the contralateral, nonsectioned hemisphere, and the total number of cells across all functional areas in the sectioned hemisphere. These correction factors were of 1.466, 1.426, 1.480, and 1.320 across the four mice analyzed, respectively.

Since functional areas were dissected by matching each dissected piece of cortex to the corresponding boundaries in the Franklin and Paxinos (2007) atlas, we made no attempt to measure their individual surface areas and volumes. Conforming each portion of cortex to the area and volume in the corresponding images in the atlas constrains experimental variation by applying the same pattern to each individual cortex. Because of this, we chose to use surface areas and volumes from the atlas, instead of measuring actual surface areas and volumes of the various dissected pieces of cortex. The use of these measurements from the atlas is further warranted by a recent MRI study of 18 mice that showed that the variation in cortical architecture in the C57BL mouse is negligible (Ullmann et al., 2013). We reconstructed surface areas and volumes from images of diagrams in the mouse brain atlas (Franklin and Paxinos, 2007) using the method of Cavalieri, with a grid size of 0.2 × 0.2 mm. The average thickness of the gray matter of each cortical area was then calculated by dividing the total volume by the total surface area of each functional area as displayed in the mouse brain atlas. These surface areas, volumes, and thicknesses thus refer to the fixed and stained cerebral cortex shown in the atlas. Therefore, while we analyze the variation of cell numbers in functional areas across individuals, we cannot report on the variance of surface areas and volumes of functional areas across individuals. Rather, we report relationships between average numbers of cells in each area and its surface area or volume as estimated from the atlas. Results are also reported as gray scales on a flat representation of the cerebral cortex drawn from all coronal sections in the Franklin and Paxinos (2007) atlas, accounting for the particular intervals between sections.

## Results

Across all cortical areas of a single cortical hemisphere of C57Bl/6J mice, excluding the hippocampus and amygdala, we found on average a total of 5.05 ± 0.82 million neurons and 6.64 ± 0.49 million other cells. These numbers closely match the numbers obtained previously in the cerebral cortex of Swiss mice (in the two hemispheres, including the hippocampus: 13.7 million neurons and 12.1 million other cells; Herculano-Houzel et al., 2006).

**Figure 1:**
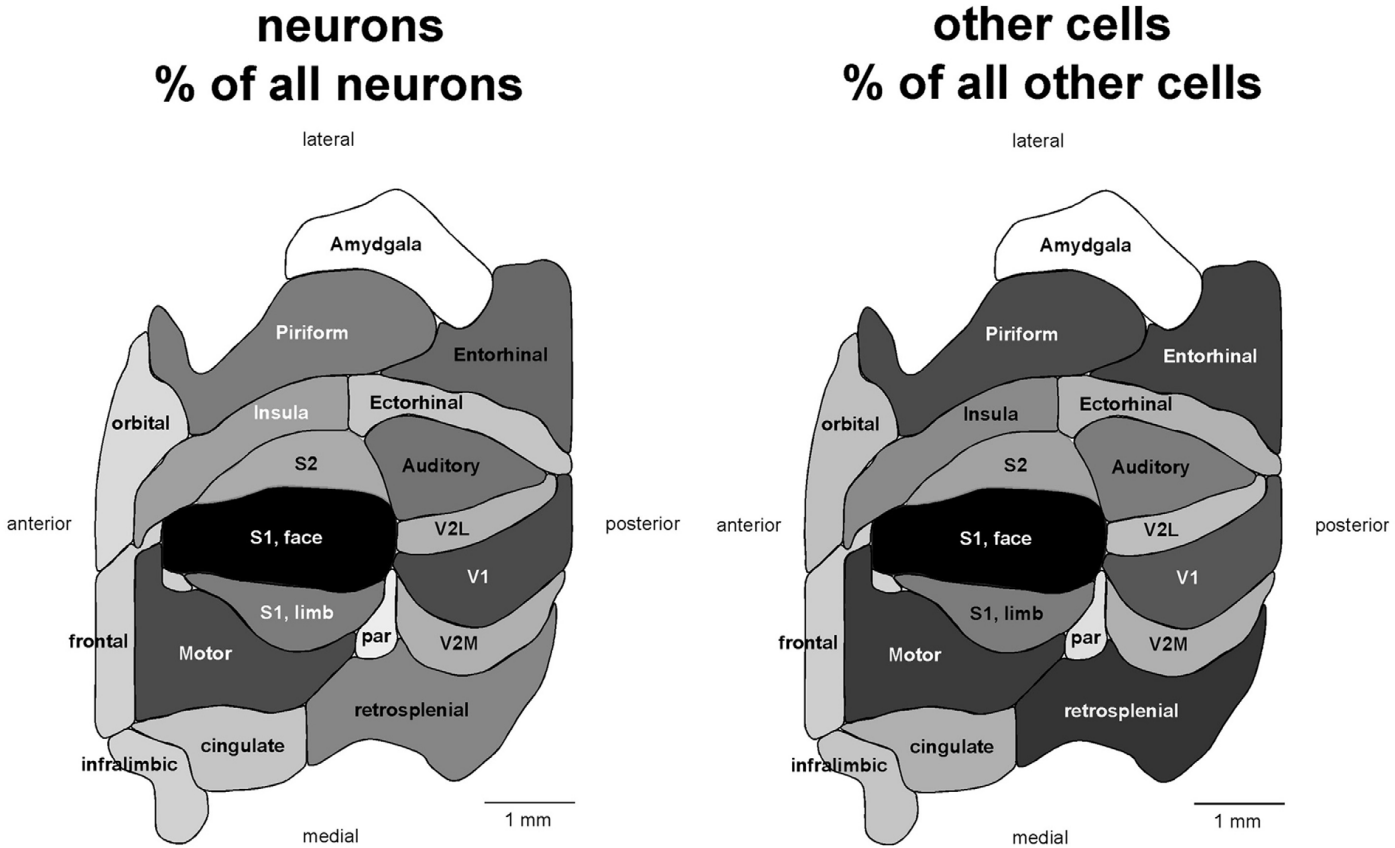
**Distribution of absolute and relative numbers of neurons (left) and other cells (right) across the 18 functional areas of the mouse cerebral cortex**. Functional areas are drawn according to their surface reconstruction from Franklin and Paxinos (2007). Gray levels represent absolute and relative numbers of neurons or other cells normalized to the maximal values (100%, that is, black, found in the S1 face area). Absolute and relative numbers of cells are found in Table 1.

Across cortical areas, neurons are heavily concentrated in the S1 face area (Figure 1, neurons), with an average of 673,919 ± 151,512 neurons, representing 13.35% of all cortical neurons. The area with the second largest number of neurons is the motor cortex (508,471 ± 74,266 neurons, or 10.07% of all cortical neurons), followed by area V1, with an average of 475,913 ± 136,968 neurons, which amounts to 9.43% of all cortical neurons (Table 1; see Table S1 for the numbers of cells in each functional area across individuals). The ensemble of all of the somatosensory areas holds 24.8% of all cortical neurons.

**Figure 2:**
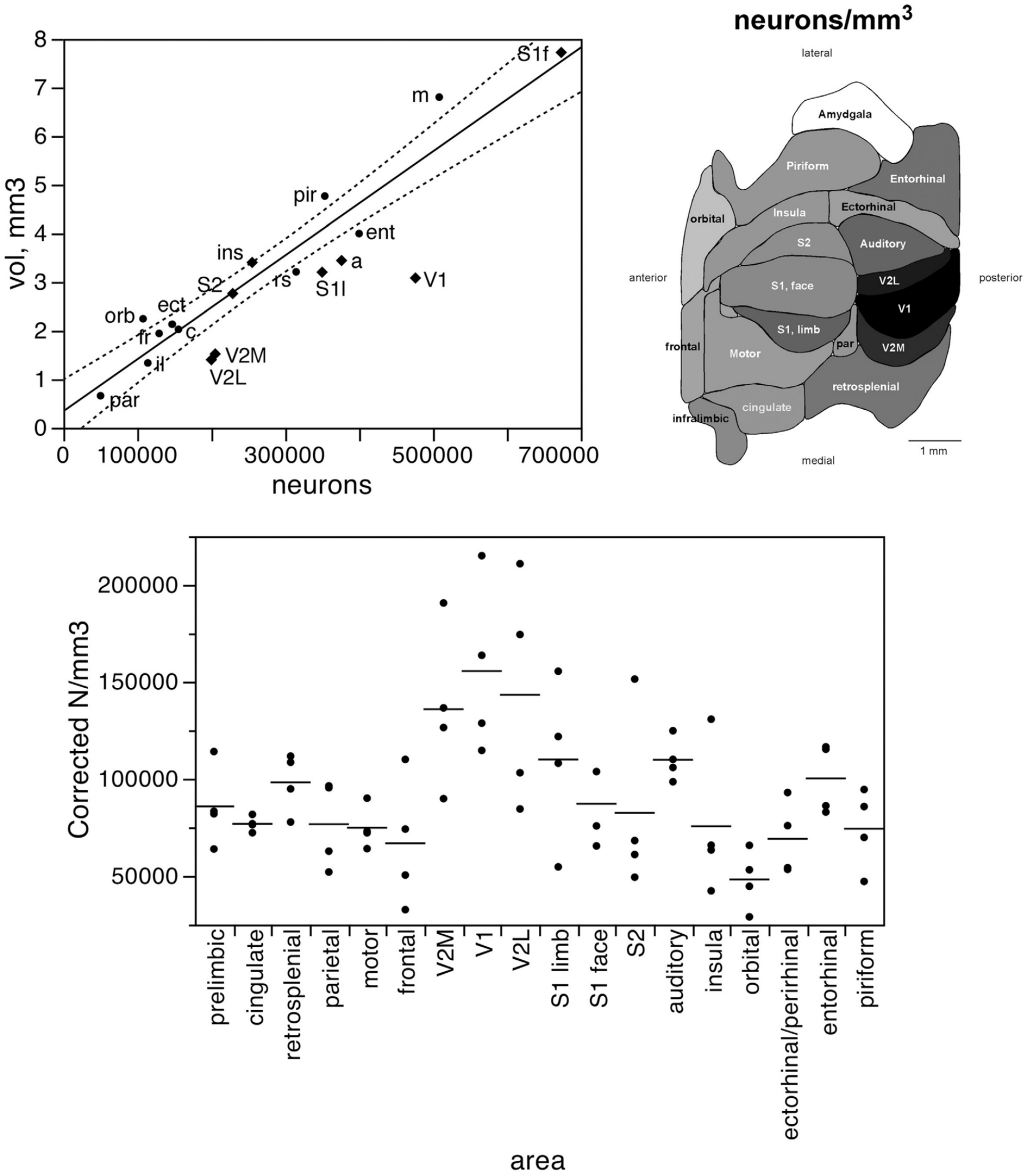
**The volume of each functional area varies as a simple shared function of the number of neurons in each area, with the exception of the visual areas**. Top left, area volume plotted as a function of the average number of neurons found in that functional area. The fitted line is the linear function that describes the entire dataset, including areas V1, V2L, and V2M, whose respective datapoints fall well outside of the 95% confidence interval (dotted lines). Top right, variation in average neuronal density (neurons/mm^3^) across functional areas. Gray levels represent neuronal density normalized by the maximal value, observed in area V1 (100%, that is, black). Bottom, variation in neuronal density across functional areas and across individuals. Horizontal lines indicate the average neuronal density found in each functional area.

If different functional areas had local, characteristic cellular properties, one should not find any systematic relationship between the volume and number of neurons across the different areas. Remarkably, we find a very good correlation between the two variables across all 18 functional areas analyzed (Spearman correlation, ρ = 0.866, *p* < 0.0001; Figure 2). Moreover, the volume of each cortical area is best described as a simple linear function of its number of neurons (all areas, *r*^2^ = 0.903, *p* < 0.0001) rather than as a power function (*r*^2^ = 0.786, *p* < 0.0001, exponent 0.814 ± 0.102). The visual areas constitute a notable exception, as V1, V2L, and V2M fall far below the confidence intervals of the linear fit, with a far smaller volume than expected for their number of neurons (Figure 2, top left). This suggests that while neuronal density does not vary systematically with area volume in non-visual areas, neuronal densities are higher than average in the visual areas compared to others.

Indeed, the visual areas have average neuronal densities of well over 100,000 neurons/mm^3^, while densities in the other areas vary from nearly 50,000 neurons/mm^3^ to around 100,000 neurons/mm^3^ (Table 1; Figure 2, bottom). In agreement with the distribution of neurons across cortical volumes being best described as linear, there is no significant correlation between neuronal densities and the volume of the non-visual area (Spearman correlation, ρ = 0.282, *p* = 0.3083). Even though V1 is the cortical area with the largest neuronal density (155,426 neurons/mm^3^), it does not have the largest number of neurons amongst cortical areas. This discrepancy shows that the number of neurons in each cortical area is also not a function of its neuronal density (Spearman correlation, ρ = 0.412, *p* = 0.0895). Although anterior areas as a whole have smaller neuronal densities than posterior areas, neuronal density does not vary as a simple rostrocaudal gradient, given that the areas both medial and lateral to visual cortex, and in particular the ectorhinal cortex, have small, average neuronal densities comparable to more anterior areas (Figure 2, top right).

**Figure 3:**
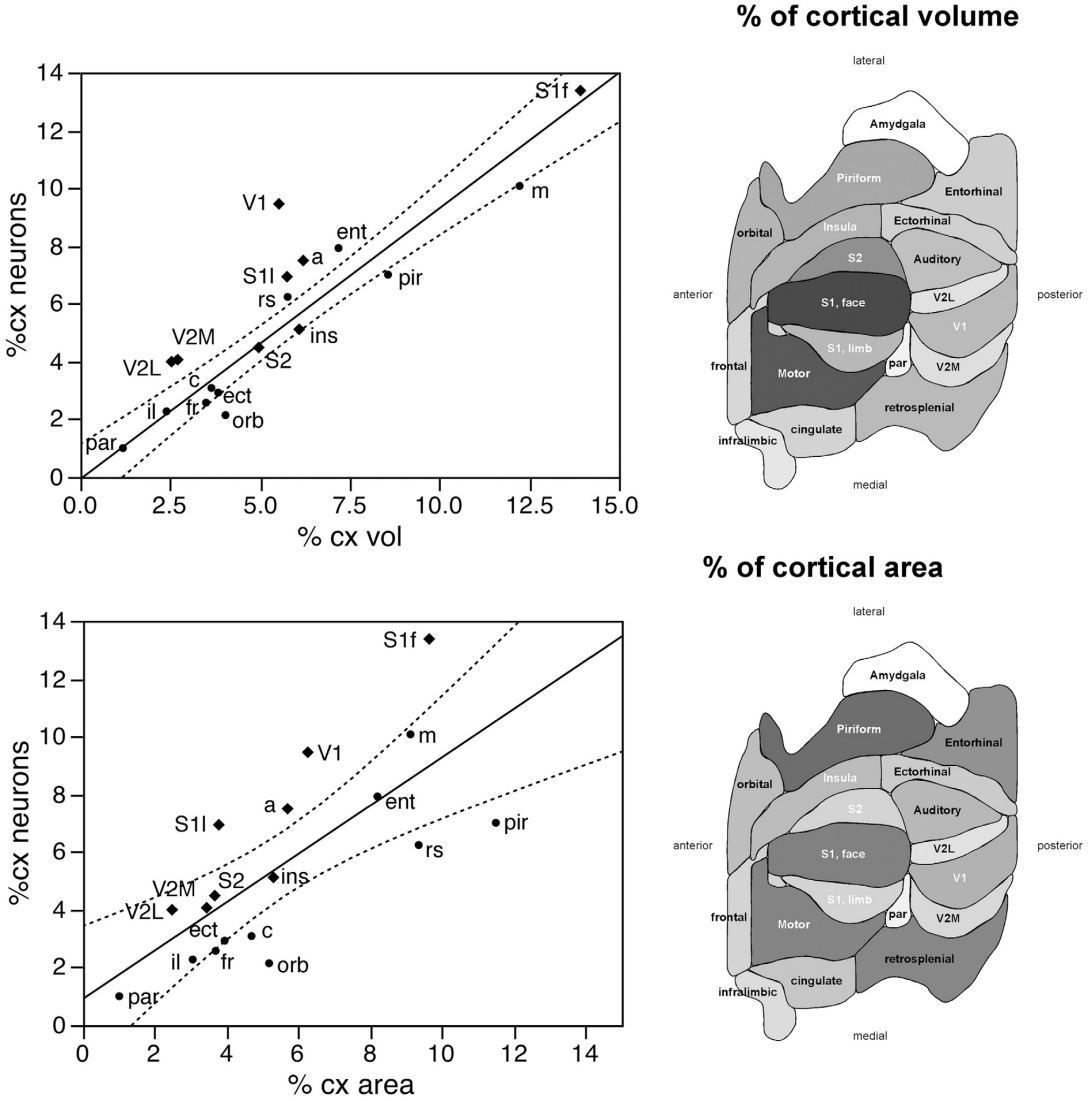
**The relative number of cortical neurons found in each functional area correlates well with the relative volume of each area, but poorly with the relative surface area of each functional area**. Top left, relative number of cortical neurons in each area plotted as a function of the relative volume of that functional area. The fitted line is the linear function that describes the entire dataset, including areas V1, V2L, and V2M, whose respective datapoints fall well outside of the 95% confidence interval (dotted lines). Top right, variation in cortical volume (absolute and relative) across functional areas. Gray levels represent normalized cortical volume. Bottom left, relative number of cortical neurons in each area plotted as a function of the relative surface area of that functional area. The fitted line is the linear function that describes the entire dataset, with the 95% confidence interval indicated by the dotted lines. Bottom right, variation in cortical surface area (absolute and relative) across functional areas. Gray levels represent normalized cortical surface.

Taken together, these findings indicate that, despite small local differences in neuronal density across the non-visual areas, neurons are distributed as a common function, with small, non-systematic variations, across the non-visual portion of the cortical volume, though concentrated at higher densities in the visual areas (V1, V2L, and V2M). If this were the case, then the fraction of cortical neurons contained in each functional, non-visual cortical area should vary linearly with the fractional volume of the respective areas. Indeed, excluding the 3 visual areas from the analysis, we find a very good linear fit between the percentages of cortical neurons and of cortical volume across functional areas (*r*^2^ = 0.910, *p* < 0.0001; Figure 3, top). The visual areas are clear outliers to the relationship: V1, which concentrates 9.43% of all cortical neurons, occupies only 5.51% of the cortical volume, while V2M and V2L, which concentrate about 4% of all cortical neurons in each, occupy 2.71% and 2.51% of the cortical volume (see Table 1).

**Figure 4:**
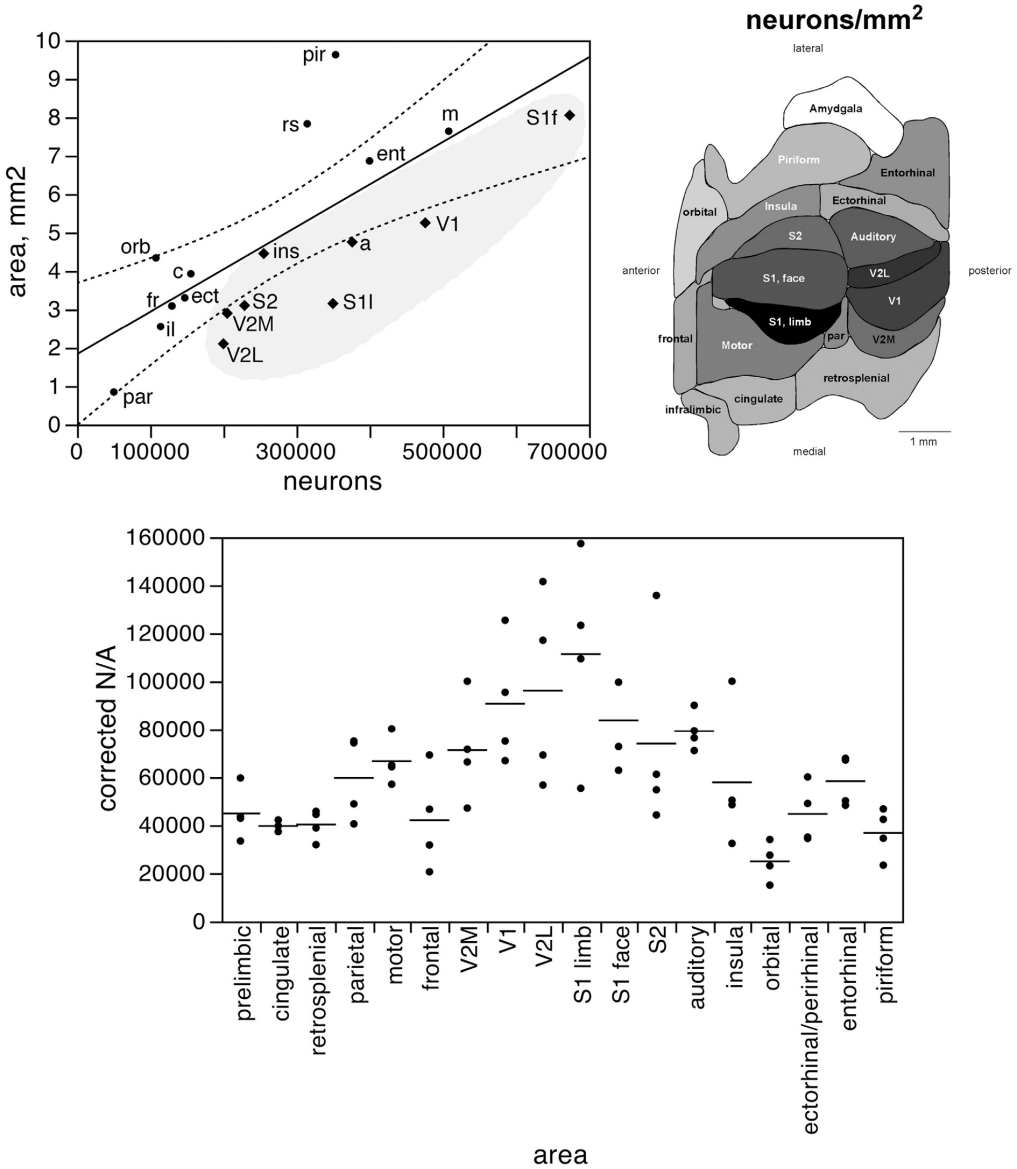
**The surface area of each functional area is not tightly correlated to the number of neurons in each area**. Top left, surface area of each functional area plotted as a function of the average number of neurons found in that functional area. The fitted line is the linear function that describes the entire dataset, with the 95% confidence interval indicated by the dotted lines. Top right, variation in average surface density of neurons (neurons/mm^2^, or N/A) across functional areas. Gray levels represent surface density of neurons normalized by the maximal value, observed in area S1limb. Bottom, variation in surface density of neurons (neurons/area, or N/A, in neurons/mm^2^) across functional areas and across individuals. Horizontal lines indicate the average surface density of neurons found in each functional area.

In contrast to the tight distribution of cortical volume as a function of numbers of neurons across non-visual areas, the relationship between surface area of individual areas and the number of neurons in them is much looser (linear fit, *r*^2^ = 0.538, *p* = 0.0011; Figure 4, top), which suggests that while neurons are distributed homogeneously per non-visual cortical volume, they are not distributed equally per cortical surface area. In line with this observation, there is a poor linear fit between the percentage of cortical neurons located in each cortical area and the percentage of cortical surface in that area (*r*^2^ = 0.517, *p* = 0.0005; Figure 3, bottom). Additionally, we find that sensory areas are located above the fit, that is, they hold a larger percentage of cortical neurons than expected from their surface area.

The distribution of neurons beneath the cortical surface, therefore, varies according to rules that differ among functional areas. Indeed, we find that N/A varies 4.5-fold across functional areas, from 24,891 ± 7,973 N/mm^2^ in the orbital cortex to 111,319 ± 42,427 N/mm^2^ in the S1 limb area, with the highest values of N/A all found in the sensory areas (in this order: S1 limb area, V2L, V1, S1 face area, auditory cortex, S2, and V2M; see Table 1; Figure 4, bottom).

**Figure 5:**
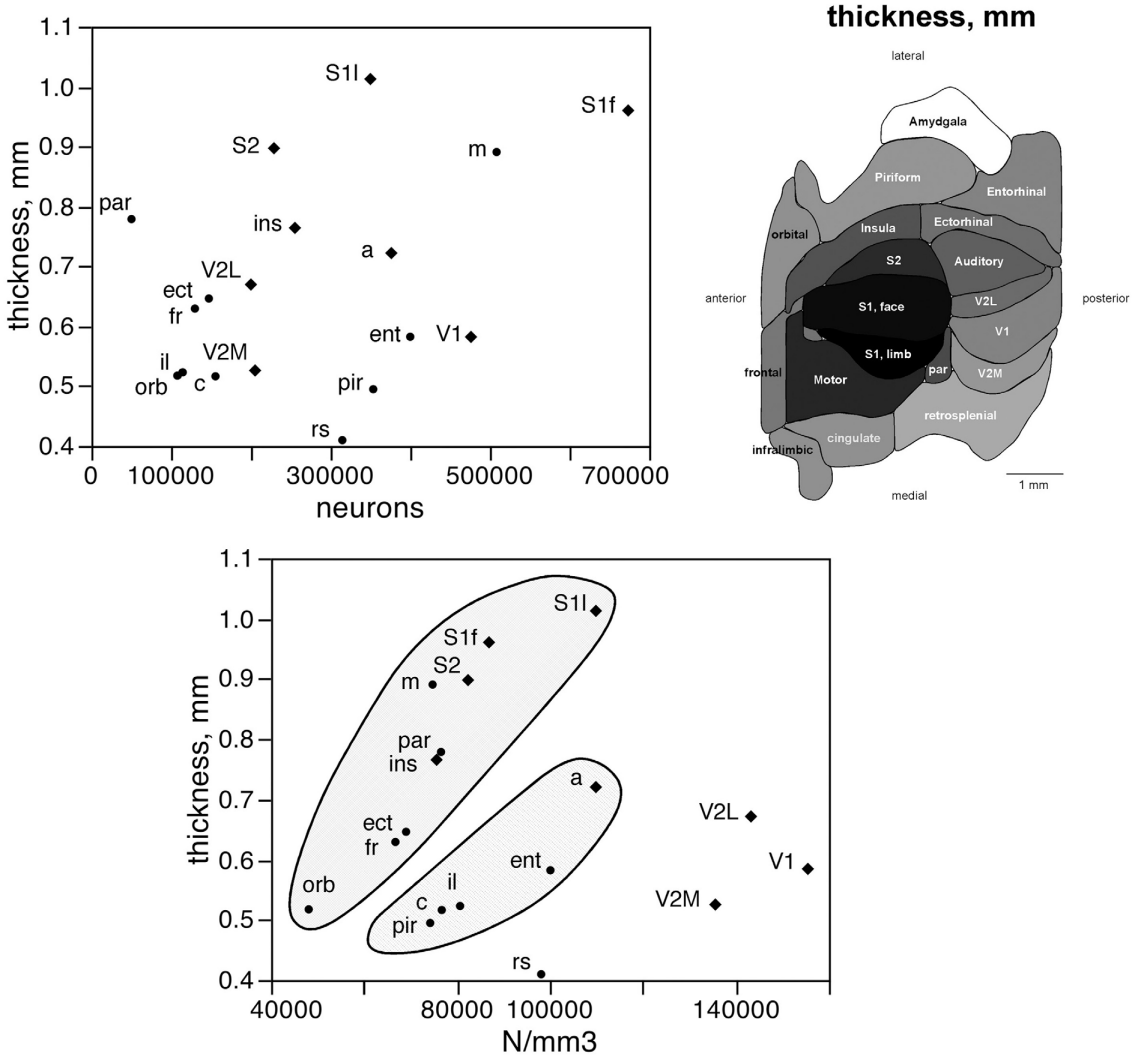
**Cortical thickness is not a simple function of the number of neurons across functional areas**. Top left, average thickness of each functional area plotted as a function of the average number of neurons found in that functional area. There is no significant correlation between the two variables (*p* = 0.2798). Top right, variation in average thickness of the gray matter of each functional area. Gray levels represent average thickness normalized by the maximal value, observed in area S1limb. Bottom, average thickness of each functional area plotted as a function of the average neuronal density in that functional area. Notice that datapoints segregate into three clusters (indicated); within each cluster, variations in cortical thickness are tightly correlated to variations in neuronal density across functional areas.

**Figure 6:**
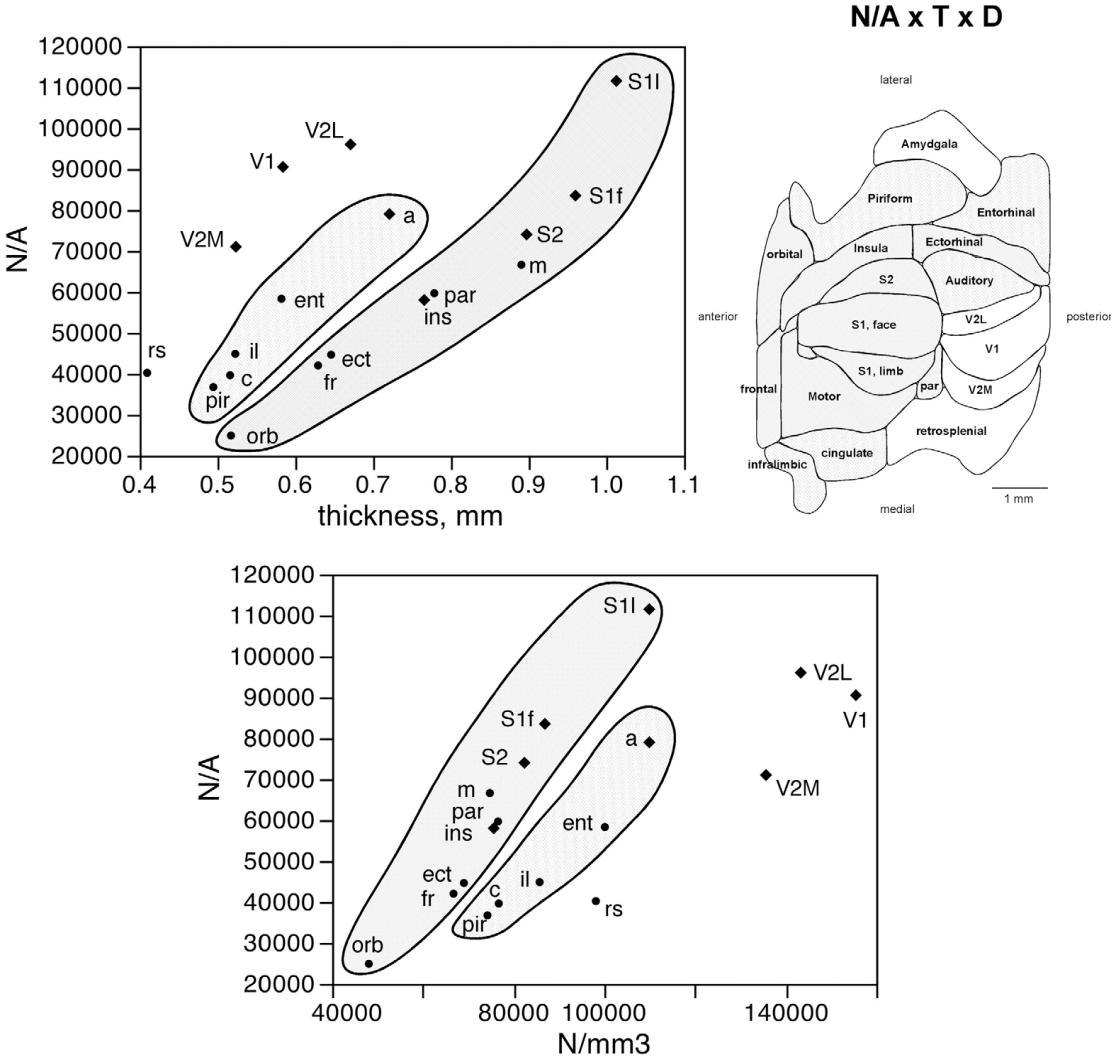
**Surface density of neurons (N/A) varies with cortical thickness and neuronal density in different ways across three cortical zones**. Top left, average surface density of neurons (N/A, in neurons/mm^2^) in each functional area plotted as a function of the average thickness of the gray matter in that functional area. Datapoints segregate into three clusters (indicated); within each cluster, variations in N/A are tightly correlated to variations in cortical thickness across functional areas. Top right, shading indicates the clustering of functional areas into the three zones indicated in the graphs according to the relationships between N/A and cortical thickness or neuronal density. Bottom, average surface density of neurons (N/A, in neurons/mm^2^) in each functional area plotted as a function of the average neuronal density in that functional area. Notice the segregation of the datapoints into the same three clusters (indicated); within each cluster, variations in N/A are tightly correlated to variations in neuronal density across functional areas.

The number of neurons in a given cortical area, N, amounts to the product of the surface of the cortical area (A), the average thickness of the gray matter of that area (T), and the average neuronal density in that area (D), such that *N* = A × T × D. Given that the product of A and T amounts to the volume of that cortical area, the finding that neurons are distributed homogeneously per cortical volume (except in the visual areas) but not per area suggests that cortical thickness varies heterogeneously across cortical areas, without a systematic relationship with *N*. Indeed, we find no significant correlation between cortical thickness and the average number of neurons across the 18 functional areas analyzed (Spearman correlation, ρ = 0.269, *p* = 0.2798; Figure 5, top). This indicates that cortical thickness is a locally determined property of cortical areas. Across all areas, there is a significant correlation between T and D (Spearman correlation, ρ = 0.746, *p* = 0.0004; Figure 5, bottom). Interestingly, however, the different functional areas seem to segregate into three clusters, or zones: one consisting of dorsal and anterior structures, one consisting of the medial and lateral border structures, and one consisting of the posterior structures (visual areas and retrosplenial cortex). Within each of these zones, T varies in strong correlation with D (Figure 5, bottom: anterior/dorsal zone, ρ = 0.782, *p* = 0.0075; medial/lateral zone, ρ = 1.000, *p* < 0.0001; posterior zone, ρ = 0.800, *p* = 0.2000). Given this zone-specific correlation between T and D, and because N/A = T.D, we find that N/A varies concertedly with both T and D, but again in different manner across the three zones: anterior/dorsal (correlation coefficients, N/A × T, 0.957; N/A × D, 0.980), medial/lateral (N/A × T, 0.989; N/A × D, 0.972), and posterior (restrosplenial/visual areas; N/A × T, 0.968; N/A × D, 0.945; Figure 6). Notice that the distributions of neuronal density (neurons/mm^3^) and N/A (neurons/mm^2^) across cortical areas do not seem to match (Figures 2, 4). Because of the zone-specific relationships between T and D, for a same neuronal density the anterior areas (checked zone in Figure 6) are found to have a larger number of neurons per mm^2^ of cortical surface than medial/lateral areas (dashed zone in Figure 6). These findings suggest that the distribution of neurons under the surface area of the mouse cerebral cortex is divided into three different zones: dorsal/anterior, medial/lateral, and posterior.

**Figure 7:**
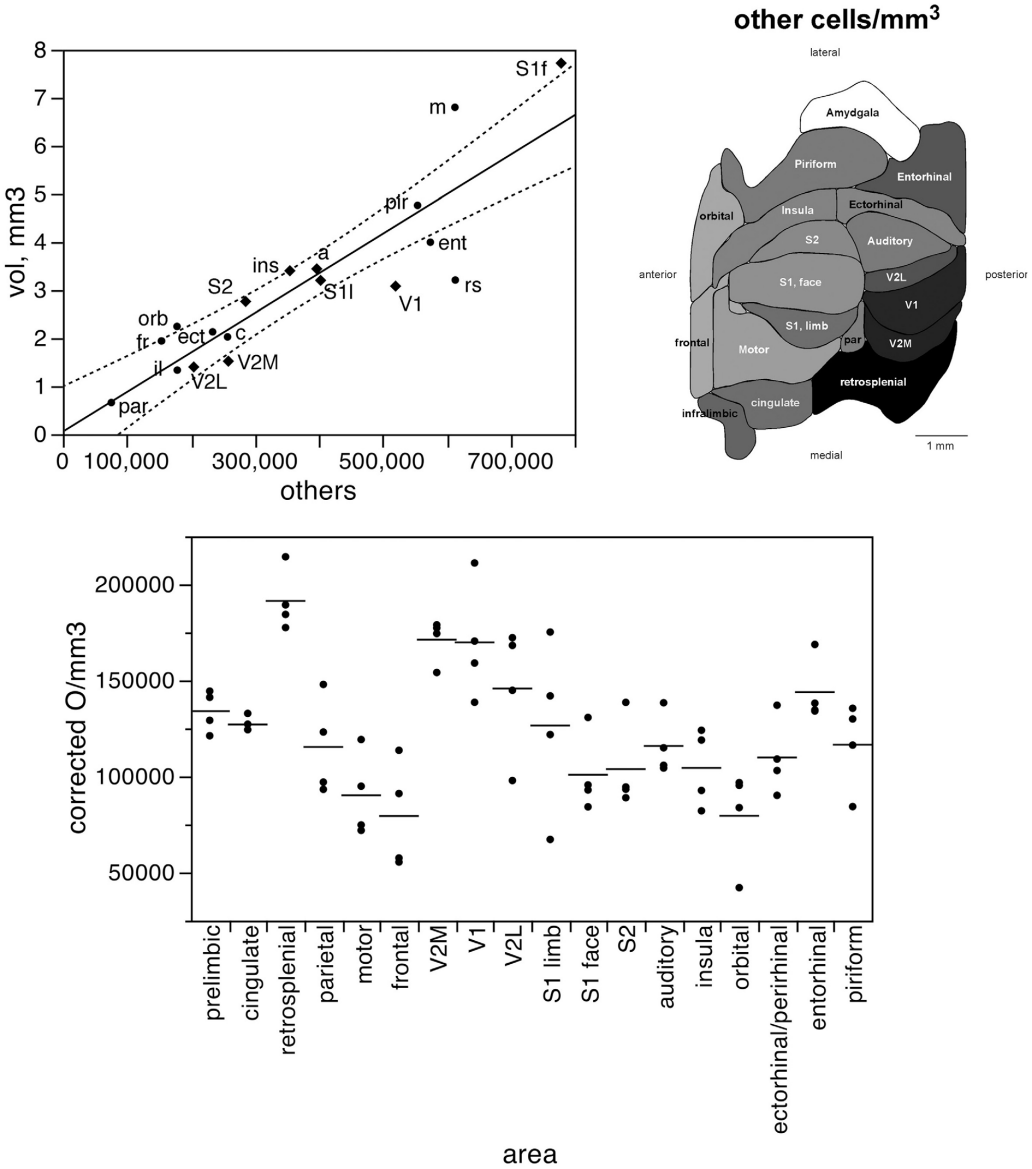
**The volume of each functional area varies as a simple shared function of the number of other cells in each area**. Top left, area volume plotted as a function of the average number of other cells found in that functional area. The fitted line is the linear function that describes the entire dataset, and the dotted lines indicate the 95% confidence interval of the fitted function. Top right, variation in average other cell density (other cells/mm^3^) across functional areas. Gray levels represent neuronal density normalized by the maximal value, observed in the retrosplenial cortex. Bottom, variation in other cell density across functional areas and across individuals. Horizontal lines indicate the average other cell density found in each functional area.

**Figure 8:**
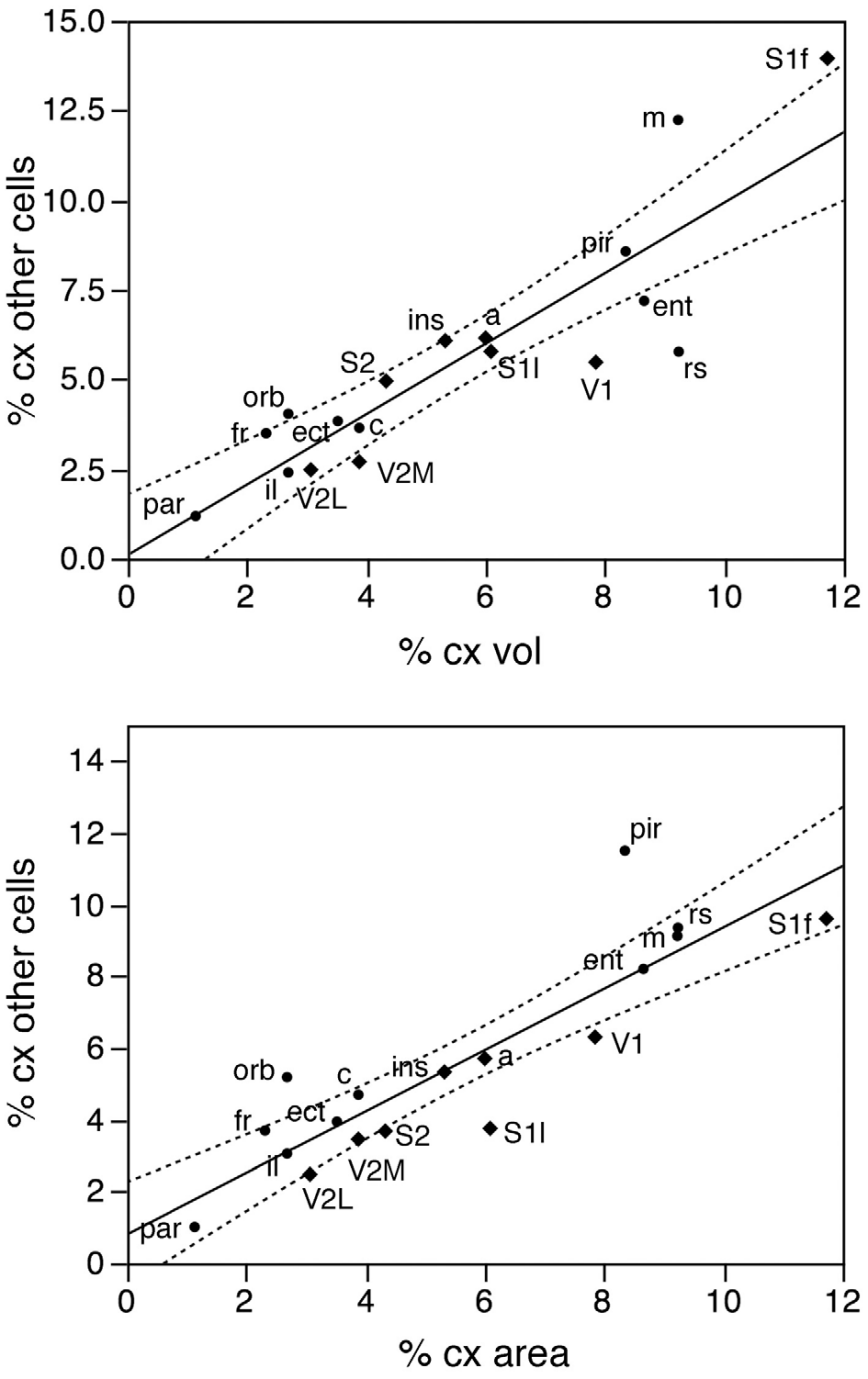
**The relative number of cortical other cells found in each functional area correlates well with both the relative volume and the relative surface area of each functional area**. Top, relative number of cortical other cells in each area plotted as a function of the relative volume of that functional area. Bottom, relative number of cortical other cells in each area plotted as a function of the relative surface area of that functional area. The fitted lines are the linear functions that describe the entire dataset; the 95% confidence intervals are indicated by the dotted lines.

In contrast to the two-zone distribution of neurons per volume and the three-zone distribution of neurons per surface area, we find a single, linear pattern of distribution of other cells (presumed to be mostly glial cells) across the volume of all functional areas (Figure 7, top; *r*^2^ = 0.775, *p* < 0.0001), with no correlation between the density of other cells and cortical volume (ρ = −0.199, *p* = 0.4282). In support of a common distribution of other cells across cortical volumes, the percentage of all-cortical other cells situated in each functional area is a direct linear function of relative cortical volume per area (Figure 8, top; *r*^2^ = 0.775, *p* < 0.0001). In contrast to the uneven distribution of neurons under the cortical surface, however, the percentage of all other cells in the cortex that are situated in each functional area is an equally good linear function of the relative surface area of each functional area (Figure 8, bottom; *r*^2^ = 0.791, *p* < 0.0001). These findings suggest that other cells are distributed in a common manner throughout the mouse cerebral cortex, irrespective of local variations in the distribution of neurons under the cortical surface.

**Figure 9:**
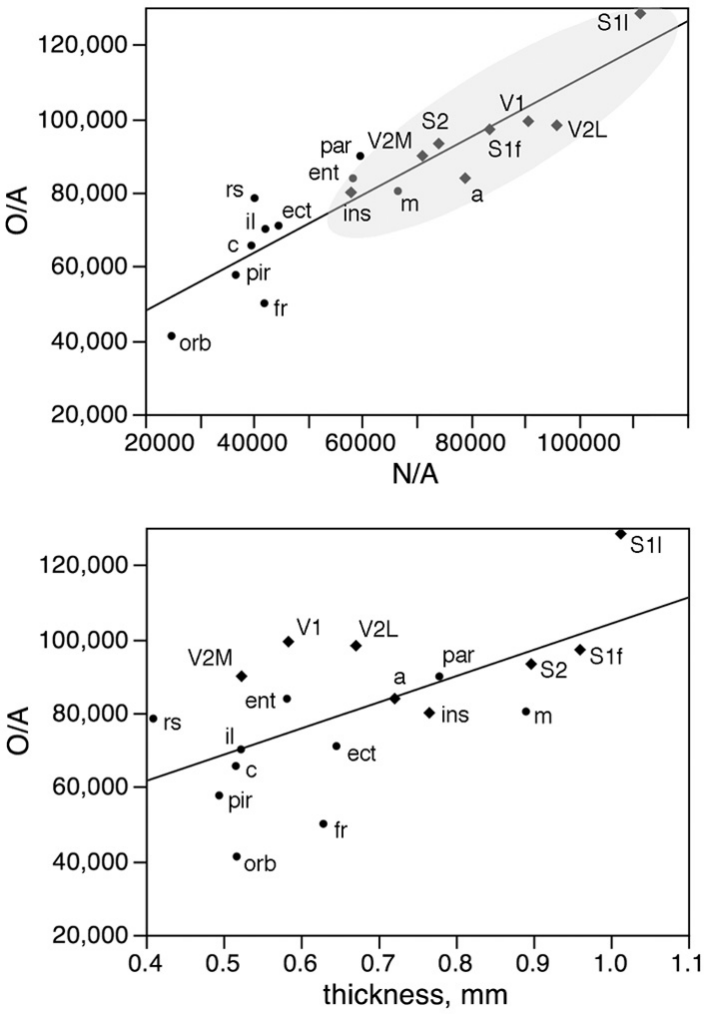
**Local variations in the distribution of other cells under the cortical surface (O/A) are tightly linked to local variations in the distribution of neurons under the cortical surface (N/A) but not to variations in cortical thickness**. Top, other cells per mm^2^ of cortical surface (O/A) in each area plotted as a function of the number of neurons per mm^2^ in the same area (N/A). Bottom, other cells per mm^2^ of cortical surface (O/A) in each area plotted as a function of thickness of the grey matter in the same area. The fitted lines are the linear functions that describe the entire dataset.

In fact, local variations in the distribution of other cells under the cortical surface (O/A) are tightly linked to local variations in the distribution of neurons under the cortical surface (N/A) as a linear function (*r*^2^ = 0.842, *p* < 0.0001; Figure 9, top), but only poorly to local variations in cortical thickness (*r*^2^ = 0.383, *p* = 0.0062; Figure 9, bottom). The correlated variations in O/A and N/A can be explained by the correlation between other cell density and neuronal density across functional areas (see below).

**Figure 10:**
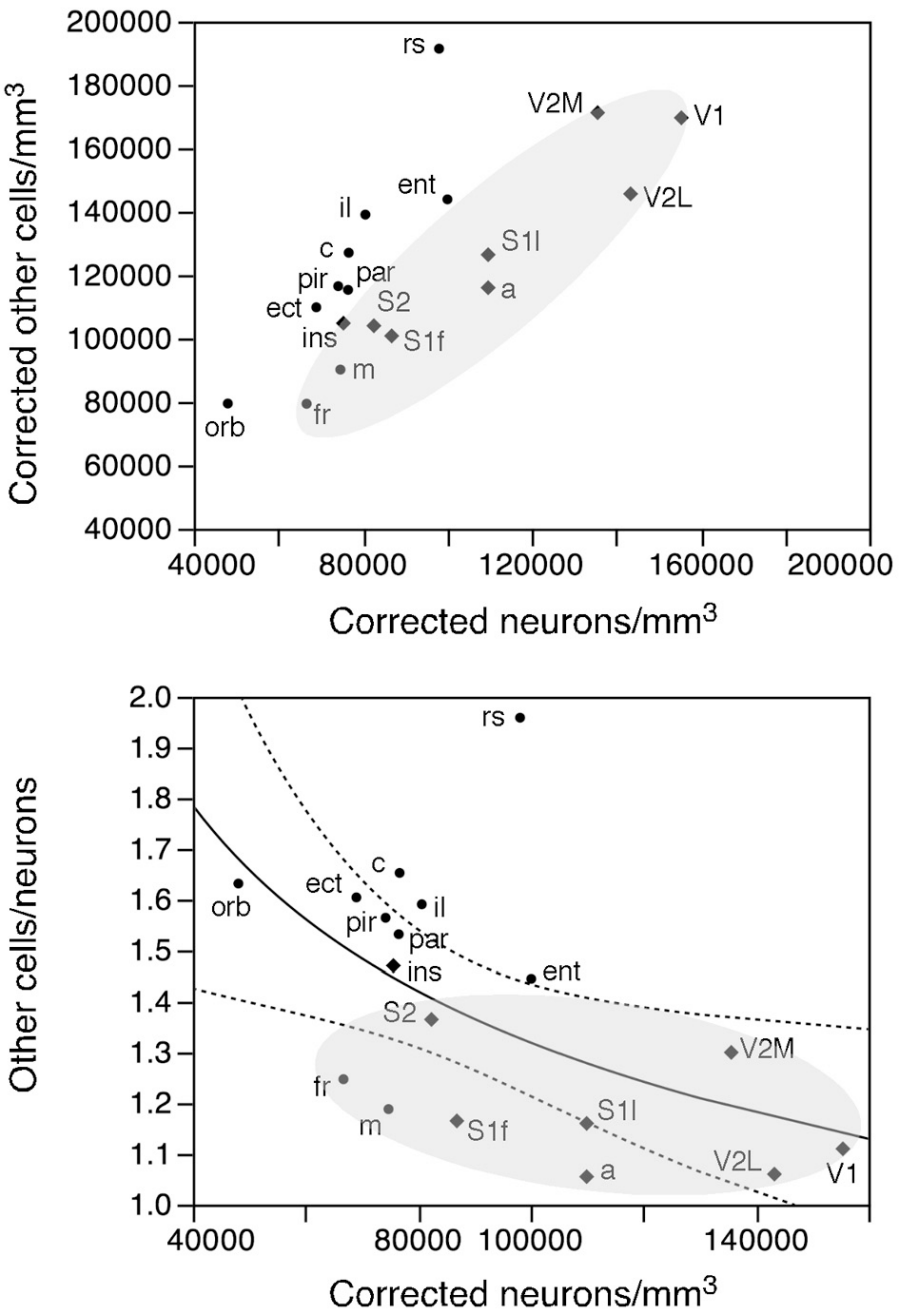
**Rules governing the distribution of other cells across cortical areas**. Top, average density of other cells in each area plotted as a function of the average density of neurons in that functional area. There is a tight correlation between the two densities across functional areas divided into two clusters: one including the sensory and motor areas and motor cortex (shaded area) and another including the remaining cortical areas. Bottom, ratio between other cells and neurons (O/N) plotted as a function of the average neuronal density in each functional area. The fitted line is the power function of exponent −0.330 that describes the entire dataset.

Like neuronal densities, the density of other cells is not homogeneous, varying by a factor of 2.4 across functional areas (Figure 7, bottom). Except for the high density of other cells in the retrosplenial cortex, the pattern of variation in other cell density resembles that of neuronal density. Indeed, densities of neurons and of other cells vary in a concerted fashion (Figure 10, top; ρ = 0.740, *p* = 0.0004), although in seemingly two different clusters: one including the sensory areas, motor, and frontal cortex (Figure 10, top, shaded areas: ρ = 0.915, *p* = 0.0002), and another for the remaining cortical areas (ρ = 0.952, *p* = 0.0003). Thus, sensory areas and the motor and frontal cortices have smaller densities of other cells than do other cortical areas of similar neuronal densities.

As a final test of our hypothesis that other cells are distributed in a common fashion across the cortical sheet, we examined the relationship between the O/N (other cells/neurons) ratio, which approximates the glia/neuron ratio, and neuronal densities across functional areas. We find that the O/N ratio varies 2-fold, between 1.062 in V1 and 1.959 in the retrosplenial cortex, and, as could be predicted from the smaller other cell densities in the sensory areas and motor and frontal cortices compared to other areas, the O/N ratio is smaller in these than in other parts of the mouse cerebral cortex (shaded area in Figure 10, bottom). Still, the variation across all functional areas is significantly related to variations in neuronal density by a power function of negative exponent (−0.330 ± 0.125, *r*^2^ = 0.302, *p* = 0.0181; Figure 10, bottom).

## Discussion

Our analysis of the distribution of neurons across functional areas of the mouse cerebral cortex shows that most neurons are concentrated in the primary somatosensory cortex, which contains 25% of all cortical neurons, followed by the motor cortex, with 10% of all neurons, and the primary visual cortex, with 9%. This distribution is consistent with a predominance of somatosensory function in mouse behavior (Fox, 2008). As a whole, the primary sensory areas in the mouse hold 62% of all cortical neurons. In contrast, the purely associative frontal areas (infralimbic, cingulate, and frontal cortex) together comprise 8% of all cortical neurons. This is surprisingly similar to the finding that the human prefrontal cortex, defined as all cortex anterior to the corpus callosum (as in Sansom and Livesey, 2009; Schoenemann et al., 2005, and comprised of purely associative areas), also only comprises 8% of all cortical neurons (Ribeiro et al., 2013).

The shared relationship across cortical areas between cortical volume and number of other cells indicates that the distribution of other (mostly glial) cells is homogeneous across cortical volumes, with local variations in density that are related to local variations in neuronal density. This is equivalent to the recent finding in Carlo and Stevens (2013) that the number of glial cells per mm^2^ of cerebral cortex increases with cortical thickness within and across species, that is, that glial cells are distributed homogeneously across the cortical volume, with only small local variations. In contrast to that study (which examined only four cortical areas in the mouse), however, we find a larger variation in the number of other cells per mm^2^ that is only poorly correlated with cortical thickness. Rather, from our analysis across 18 different functional areas, we find a single and very strong relationship between the local number of other cells per mm^2^ and the local number of neurons per mm^2^ across functional areas, as expected from the correlation between neuronal and other cell densities. Thus, local variations in other cell density are related to local variations in neuronal density. Further, our observation extends to different areas within the cerebral cortex our previous findings that the relationship between the O/N ratio and neuronal density is shared across different brain structures and even mammalian species, as is the relationship between neuronal and other cell densities (reviewed in Herculano-Houzel, 2011). Given that the O/N ratio is exactly the ratio between other cell density and neuronal density, and we show that these are directly related, our data disagree with the conclusion by Carlo and Stevens (2013) that local variations in the O/N ratio are driven by cortical thickness. The commonality in the distribution of other cells within the cortex and across structures and species indicates that the mechanism whereby these cells are added to the parenchyma in brain development has been preserved for at least 95 million years (Herculano-Houzel, 2012b). The common relationship between the O/N ratio and neuronal density across functional areas supports our hypothesis that glial cells are added homogeneously to brain tissue until they occupy the entire volume and reach confluency, and glia/neuron ratios are simply a consequence of the average size of the neurons in the parenchyma (that is, roughly the inverse of neuronal density). Notice that “average neuronal size” refers to the size of the entire neuronal cell, with all dendritic and axonal arbors, and not only the cell soma. Because non-neuronal density varies little in comparison to variations in neuronal density, the latter can be approximated as the inverse of average neuronal size: the smaller the average size of neurons (cell bodies and arbors), the fewer neurons will be found per volume, and therefore the smaller the neuronal density. According to our hypothesis (Herculano-Houzel et al., 2006; Herculano-Houzel, 2011), as glial cells invade the cerebral tissue postnatally, higher glia/neuron ratios are established in regions of smaller neuronal densities, which are indicative of larger average neuron sizes, such as the frontal cortex, which matches reports of neurons with larger dendritic arbors in frontal cortex than in other areas in the mouse (Benavides-Piccione et al., 2006). Conversely, lower glia/neuron ratios are established where neuronal densities are larger, indicative of smaller average neuronal sizes, such as in the occipital cortex, again in agreement with reports of smallest dendritic arbors in the occipital cortical areas (Benavides-Piccione et al., 2006).

Neurons, however, are distributed differently across visual and non-visual areas of the mouse cerebral cortex. Visual areas V1 and V2 seem to comprise a separate zone of the cortical gray matter, with larger neuronal densities than in any other functional area, while in the non-visual cortex, the volume of a functional area is a shared function of its number of neurons. This suggests that neurons are distributed in a common fashion across the non-visual volume of the cortical gray matter. This division of the cortical gray matter into two zones that are quantitatively distinct in their distribution of neurons is remarkably similar to that found in the human cerebral cortex (Ribeiro et al., 2013).

While there seem to be two cortical zones (visual and non-visual) that differ in how neurons are distributed across the cortical volume, our data suggest that the cortex can be divided into three zones that differ in how neurons are distributed underneath the cortical surface–an anterior/dorsal zone, a medial/lateral zone, and a posterior zone (corresponding to visual and retrosplenial cortices). Within each zone we find a particular relationship between the distribution of neurons under the surface (the N/A ratio, the number of neurons under 1 mm^2^ of pial surface) and neuronal density or cortical thickness, although in each of them N/A increases together with variations in neuronal density and cortical thickness across functional areas. Again, this division into three zones is remarkably similar to that found in the human cerebral cortex, in which we show that the three zones, defined by their particular rules of N/A variation, differ in their pattern of connectivity through the white matter (Ribeiro et al., 2013).

Our finding of systematic variations in the N/A ratio across functional areas of the mouse cortex is at odds with the conclusion generally drawn from the findings of Rockel et al. (1980) that neurons are uniformly distributed underneath the surface of non-visual cortical areas. In a recent recreation of that study using modern stereology methods, Carlo and Stevens (2013) analyzed numbers of neurons per mm^2^ in motor, somatosensory, parietal, and temporal areas of four different species, including mouse, and report an average N/A of 94.0 ± 10.7 neurons/mm^2^, that is, with a 95% interval of variation between 72,600 and 115,400 neurons/mm^2^. These values are squarely within the range of N/A variation observed in our present analysis. However, in the absence of a systematic analysis over the entire cortical surface, Carlo and Stevens (2013) interpret their finding of a variation in N/A of at least 50% within the mouse (see their Figure 2b) as evidence of a uniform distribution of neurons underneath the surface. Using stereology, their analysis was necessarily limited to regions where cells could be counted orthogonally to the surface. Because we count all cells underneath a given cortical surface, we did not have that limitation, and could not only examine the variation in the N/A ratio across the entire cortical surface, but also determine whether this variation was systematically related to other parameters of cortical morphology. As discussed above, we found that the N/A ratio varies systematically together with neuronal density and cortical thickness within each of three identifiable zones of the cortical surface. These findings are essentially identical to those obtained in the human cerebral cortex (Ribeiro et al., 2013). In conjunction with the by now large body of data showing variations in the N/A ratio within the entire cortical sheet (Collins et al., 2010; Cahalane et al., 2012) and across species (Haug, 1987; Stolzenburg et al., 1989; Poth et al., 2005; Herculano-Houzel et al., 2008), we consider that the distribution of neurons underneath the cortical surface must no longer be considered uniform.

It has been suggested that the cerebral cortex is organized as a single antero-posterior gradient of neuronal densities, which raises the possibility that it is formed by a single isocortex-wide mechanism that regulates numbers of neurons and their densities jointly in development (Finlay et al., 1998; Cahalane et al., 2012). We do not find evidence of such a gradient of neuronal densities in the mouse cortex, although we admit that our analysis was conducted on anatomically delimited functional areas that are not perfectly arranged along the anteroposterior axis. Still, the existence of a single mechanism that regulates neurogenesis along the entire budding cortex is contradicted by the incongruence in the distribution of neurons within the cortical volume between the visual and non-visual areas found in the present study, which in turn is consistent with the distinction between occipital (visual) and non-occipital zones of the cortex found in the human cortex (Ribeiro et al., 2013). This is in line with an earlier report of distinct cell cycle dynamics in primate striate cortex (Dehay et al., 1993), which is more consistent with a separate cortical zone of visual cortex (this study) than with a single, continuous gradient of neurogenesis along the entire cortex (Finlay et al., 1998; Cahalane et al., 2012). Our finding of two zones of different neuronal distributions indicates that in the mouse, as in the human cortex, there is not one single isocortex-wide developmental pattern, but two. We speculate that these zones correspond to those zones specified by different morphogens in the developing cerebral cortex, expressed in gradients that might be related to those found here (Bishop et al., 2000; Fukuchi-Shimogori and Grove, 2001; O'Leary et al., 2007), and which may or may not correspond to gradients of neurogenesis, but which lead to the formation of cortical zones with different relationships between cortical volume and numbers of neurons (that is, neuronal density, and therefore average neuronal cell size). FGF8 is a prime candidate molecule to cause the division of the cerebral cortex into two “occipital and non-occipital” zones, given the rostral location of its source (Bachler and Neübuser, 2001) and its suppressing role on presumptive visual areas (Fukuchi-Shimogori and Grove, 2001; Garel et al., 2003; Sansom and Livesey, 2009; Schoenemann et al., 2005; O'Leary et al., 2007). We propose that, within each zone, local regulatory mechanisms render different the relationship between neurogenesis and the average size of the resulting neurons.

We stress that a common distribution across functional areas within a cortical zone is not necessarily an even distribution of neurons. Both in mouse and in human cerebral cortices, we do find local variations in neuronal density, and those may be directly related to local variations in numbers of neurons by the very same mechanism that regulates neurogenesis, as proposed for the human cerebral cortex (Ribeiro et al., 2013). Thus, a common distribution of neurons across the volume of different functional areas means simply that, despite small variations in neuronal density, the number of neurons found in a functional area is a direct, linear function of the volume of that area—which, in turn, suggests that the mechanism by which neurons are added to the growing parenchyma is shared across non-visual areas. We also stress that the present study is limited to the average characteristics of functional areas of the cortex, such as average numbers of neurons and average neuronal density, overlooking differences across cortical layers within each functional area that define these very layers.

Notice that the two-zone scenario that we propose does not rule out a relationship between gradients in local neuronal densities and gradients in cortical neurogenesis (Finlay et al., 1998). However, while that model posits that the largest delay in neurogenesis in occipital regions leads to their large neuronal densities, the generation of larger numbers of neurons will only lead to larger neuronal densities if the newly generated neurons invade a volume that is already delimited. This is unlikely to be the case, if the cortical volume is created by the very newly generated neurons as they migrate through the cortical plate and expand it. Thus, late neurogenesis does not necessitate that the resulting neurons will be smaller and accumulated in larger densities. The distinct relationship between local gray matter volumes and numbers of neurons in the visual (occipital) cortex calls for the recognition that this is a distinct cortical zone, which is thus likely formed as the result of a distinct developmental program, one that possibly leads to the generation of neurons that are, on average, smaller than in the other cortical zone.

If different non-visual functional areas share a common distribution of neurons across the cortical volume, then what delimits each functional area and determines how many neurons are assigned to it? We envisage that the ventricular zone is initially differentiated by morphogens into two quantitatively different zones, prospective visual (occipital) and non-visual, and newborn neurons are first distributed equally within each zone forming its cortical parenchyma (with local variations in density within each possibly according to a gradient in the timing of neurogenesis or other factors). Next, we posit that this parenchyma (whose local volume is a common function of the local number of neurons, even if there are local variations in neuronal density) becomes segregated into functional areas as they form their connectivity, that is, as both afferent and efferent connections are formed and shaped. It is thus simply the distribution of afferents and efferents that makes each area a functionally specialized and recognizable area. It is unclear at this point how local differences in cytoarchitecture develop across functional areas, but the shared volumetric neuronal composition across regions as different as sensory, motor and associative areas within the non-visual cortex points to again to an instructive role of connectivity. Our findings thus support a combination of the “protomap” and “protocortex” models of specification of cortical areas (Dehay and Kennedy, 2007), one in which two zones are specified in the gray matter that differ in how neurons are added to the developing cortex, followed by functional regionalization possibly defined both by patterns of intrinsic molecules and incoming thalamic afferents (O'Leary et al., 2007).

How could neurons be distributed commonly across the (non-visual) cortical volume, but differently along its surface? To use a culinary abstraction, we can imagine how a same volume of jam, with a similar distribution of fruit within it, could be spread more or less thinly over the surface of a larger or smaller piece of toast. Building on our findings for the human cortex that the surface distribution of neurons is related to connectivity patterns (the local fraction of neurons connected through the white matter and the average caliber of these fibers), we propose that the volume of neurons that will populate the different functional areas are spread laterally in different manners across the three zones identified here (anterior/dorsal, medial/lateral, and posterior). Incidentally, the clustered location of these zones of different patterns of connectivity through the white matter is also compatible with their specification by morphogens whose expression is limited to different regions. Thus, our study opens new venues for future studies of cortical regionalization that could relate patterns of distribution of morphogens and of thalamic afferents to the patterns of distribution of neurons across the cortical volume and surface area as well as across functional areas, as described here.

### Conflict of interest statement

The authors declare that the research was conducted in the absence of any commercial or financial relationships that could be construed as a potential conflict of interest.
