# Supplementary material for: Distribution of neurons in functional areas of the mouse cerebral cortex reveals quantitatively different cortical zones
Source: Front Neuroanat. 2013 Oct 21;7:35. doi: 10.3389/fnana.2013.00035 (PMC3800983; doi:10.3389/fnana.2013.00035)
Supplement: Supplementary file 1 [file DataSheet1.DOC]

Supplementary Table 1. Distribution of neurons across cortical areas in individual mouse brains.

| Area | Neurons | | | | Other cells | | | |
| --- | --- | --- | --- | --- | --- | --- | --- | --- |
|  | B | C | D | E | B | C | D | E |
| infralimbic | 85,138 | 111,070 | 152,077 | 109,307 | 161,638 | 172,348 | 188,323 | 192,645 |
| cingulate | 154,618 | 146,283 | 165,372 | 155,685 | 257,695 | 269,039 | 250,878 | 251,867 |
| retrosplenial | 347,882 | 358,004 | 303,962 | 249,198 | 687,481 | 591,177 | 569,238 | 607,152 |
| parietal | 41,253 | 63,379 | 34,209 | 62,740 | 61,367 | 81,004 | 63,841 | 97,310 |
| motor | 496,080 | 611,947 | 490,656 | 435,204 | 508,130 | 810,488 | 645,244 | 488,840 |
| frontal | 63,453 | 213,502 | 143,862 | 97,860 | 107,581 | 176,865 | 220,588 | 111,690 |
| V2M | 205,944 | 287,658 | 190,688 | 135,406 | 263,176 | 267,590 | 269,962 | 232,544 |
| V1 | 500,772 | 658,299 | 350,809 | 393,773 | 486,945 | 646,491 | 424,341 | 521,978 |
| V2L | 243,445 | 294,331 | 117,872 | 143,897 | 234,837 | 240,419 | 202,178 | 136,604 |
| S1 limb | 344,510 | 495,544 | 174,205 | 388,311 | 388,490 | 452,746 | 214,295 | 558,789 |
| S1 face | 802,151 | 801,988 | 505,908 | 585,629 | 651,021 | 1,011,068 | 718,792 | 739,323 |
| S2 | 418,018 | 168,483 | 136,215 | 188,335 | 382,784 | 261,099 | 257,835 | 245,616 |
| auditory | 378,152 | 428,874 | 338,596 | 363,825 | 395,163 | 475,745 | 358,854 | 363,825 |
| insula | 443,814 | 223,471 | 215,080 | 143,616 | 314,841 | 403,969 | 421,320 | 278,784 |
| orbital | 65,046 | 100,148 | 119,204 | 147,431 | 94,381 | 217,137 | 187,896 | 213,919 |
| ectorhinal | 161,628 | 115,482 | 113,628 | 197,980 | 192,045 | 219,628 | 232,322 | 292,070 |
| entorhinal | 330,938 | 465,098 | 460,175 | 343,865 | 537,667 | 673,919 | 551,775 | 535,585 |
| piriform | 449,775 | 407,939 | 224,387 | 332,269 | 618,573 | 401,316 | 645,113 | 553,781 |

Numbers are corrected to compensate for losses due to sectioning and reflect total numbers of neurons or other cells per functional area in one cortical hemisphere of each of four individual mice (B, C, D and E).
